# Prevalence and antimicrobial resistant *Campylobacter* spp. in broiler chicken carcasses and hygiene practises in informal urban markets in a low-income setting

**DOI:** 10.1371/journal.pone.0318516

**Published:** 2025-01-30

**Authors:** Pius Okello, Olivia Graaf Bjöersdorff, Ingrid Hansson, Sofia Boqvist, Joseph Erume

**Affiliations:** 1 College of Veterinary Medicine, Animal Resources and Biosecurity, Makerere University Kampala, Kampala, Uganda; 2 Department of Animal Biosciences, Swedish University of Agricultural Sciences, Uppsala, Sweden; Bangladesh Agricultural University, BANGLADESH

## Abstract

Campylobacteriosis is one of the most commonly reported foodborne diseases and is of particular importance in low-income countries. More data is needed to better understand the epidemiology of *Campylobacter* spp. in food sold at informal markets, where most people in low-income countries buy their food. This study aimed to determine the prevalence and antimicrobial resistance (AMR) of *Campylobacter* spp. among broiler chicken carcasses sold at informal urban markets in Uganda and to gain more knowledge about hygienic handling practices and awareness of foodborne bacterial diseases among the market vendors. In total, 120 broiler chicken carcasses from 30 different markets were analysed using ISO 10272 and confirmed by PCR. AMR analyses were performed using the disc diffusion test. Epidemiological data on food safety practices and awareness was collected from the vendors using a questionnaire. *Campylobacter* spp. was isolated from 66% (79/120) of the carcasses; 32% were *C*. *jejuni*, 14% were *C*. *coli* and 54% comprised of a mixture of both species. All *C*. *jejuni* isolates showed resistance to tetracycline, 88% to ciprofloxacin and 28% to erythromycin. Of the *C*. *coli* isolates, 82% showed resistance to tetracycline, 73% to erythromycin and the quinolones ciprofloxacin and nalidixic acid. More than half of the vendors had heard about food-borne illnesses, but none knew about *Campylobacter* spp., and the knowledge regarding hygienic practices was low. These data calls for urgent interventions to improve food safety, protect the public from foodborne illness, and prevent the spreading of AMR.

## Introduction

Campylobacteriosis is one of the most commonly reported foodborne zoonotic diseases of high public health importance [1], for example, within the European Union and Southeast Asia [2, 3]. Within the EU, surveillance programmes for *Campylobacter* spp. in broilers are in place in several countries [4], but this is an exception globally. Campylobacteriosis is of particular importance in low- and middle-income countries (LMIC) in Africa [5, 6] as it has been reported that campylobacteriosis causes the second highest burden of food-borne disease on the African continent (after non-typhoidal salmonellosis) as measured by Disability Adjusted Life Years (DALY) [1, 7]. There is lack of information on campylobacteriosis occurrence from many African countries, for example, there is only one published paper from Uganda about *Campylobacter* spp. among children with acute diarrhea attending one of the major hospitals in the capital [8]. Campylobacteriosis in humans is characterised by symptoms such as diarrhoea, fever, and vomiting. It can, in rare cases, lead to post-infectious sequelae such as Guillain-Barre and irritable bowel syndromes [9].

*Campylobacter* spp. is frequently isolated from raw meat products, mainly from poultry [3]. There is strong evidence that poultry and poultry products are the primary sources of human campylobacteriosis [7, 9], for example, when handling, preparing and consuming contaminated chicken. In most parts of the world, poultry is frequently colonised with *Campylobacter* spp. in the intestines without showing clinical symptoms [10]. The bacteria may contaminate the carcass during slaughter and further processing, enabling spread and cross-contamination in the poultry value chain [10]. This is particularly important as animal-sourced food (ASF) is an essential source of high-quality nutrients, especially for children and pregnant women in LMIC [7, 11]. Several food safety challenges in the broiler chicken value chain jeopardise public health in LMICs. For instance, many smallholder broiler chicken farmers in Africa sell and slaughter poultry at informal markets, which are unregulated and where food safety practices and sanitation are inadequate [12, 13]. Poor food safety-related practices have also been documented, for example, in informal public markets in Kenya and ready-to-eat markets in Uganda [14, 15]. In most LMICs, there is also rapid urbanisation, which places an enormous demand for ASF with a marked increase in consumption of broiler chicken and products thereof, which raises public health concerns as this might increase the risk of people being exposed to *Campylobacter* spp. [16].

Earlier publications from Kenya have shown high prevalences (64 and 91%) of *Campylobacter* spp. in broiler chickens and chicken carcasses [16, 17]. To the authors knowledge, there is no published article about *Campylobacter* spp. in Ugandan poultry. Other published data from the African continent (here represented by Senegal, Cameroon, Ghana and Ethiopia) have reported *Campylobacter* spp. occurrence in poultry between 21 and 93% [18–20]. The literature on the prevalence, risk factors and AMR of thermotolerant *Campylobacter* spp. in humans and food-producing animals in sub-Saharan Africa is generally scarce [21].

The emergence, spread and persistence of AMR has been declared a global public health emergency by WHO [22]. AMR is a particular challenge in LMICs, partly due to the unrestricted use of antimicrobials and poor adherence to legislation [23]. Overall, there is limited data on AMR in thermotolerant *Campylobacter* spp. isolated from the African continent, but published studies indicate high levels of resistance against antimicrobials such as fluoroquinolones, macrolides and tetracycline [21, 24–27]. One review also highlights the alarming trend of increased AMR in *Campylobacter* spp. to critically important antimicrobials, such as ciprofloxacin and erythromycin [5]. In Uganda, Veterinary services are not readily available, affordable or adhered to, leading to farmers easily accessing drugs over the counter without a prescription [28, 29]. This has resulted in imprudent use of antimicrobials to manage infectious diseases as a result of limited professional advice and as a way to compensate for poor livestock management. A similar situation has been reported in neighbouring Kenya, where poultry farmers access antimicrobials without prescription for various reasons, including growth promoters, disease treatment, and stress management [30].

The main objective of the present study was to determine the prevalence of *Campylobacter* spp. among broiler chicken carcasses sold at urban informal markets, as well as the presence of AMR in the isolated *Campylobacter* spp. strains. Another aim was to investigate hygienic practices and awareness of foodborne bacterial diseases among market vendors. These data are important for gaining a better understanding of the epidemiology of *Campylobacter* spp. in the informal broiler chicken value chain in LMIC.

## Materials and methods

### Study design and data collection

This cross-sectional study was conducted at informal markets in three urban districts (Kampala, Wakiso and Mukono) in the capital Kampala, Uganda, between February and August, 2022 and included collection of broiler chicken carcasses and interviews with market vendors. Before starting fieldwork, a visit was made to each District Veterinary Office (DVO) to introduce the study, seek their approval and plan data collection. The district extension workers in each district provided a list of all the informal markets that sold raw broiler chicken products. From these lists, ten markets per district were randomly selected, and broiler chicken carcasses were sampled from each market. The calculated sample size was 124, which would be enough to detect a prevalence of 75%, with an estimated error of 5% and a confidence level of 80% [31]. The estimated prevalence for the sampling size calculation was based on published studies from neighbouring Kenya [12, 13]. From each of the ten included markets, four broiler chicken carcasses from equal numbers of vendors were planned to be sampled. The investigating team, consisting of the first author, a market representative, and a representative from the DVO, selected vendors to represent the entire market. One broiler chicken carcass was purchased from each included vendor, aseptically packed into new and clean plastic bags, labelled, sealed and transported in cool boxes containing ice packs. The laboratory analyses were initiated at the microbiology laboratory at the College of Veterinary Medicine, Animal Resources and Biosecurity (COVAB), Makerere University, Kampala, four to six hours after sample collection.

A written questionnaire was developed to capture information on the included vendors’ knowledge and practices related to foodborne illness and AMR, focusing on the spread and mitigation of foodborne bacteria at informal markets. The hygienic practices assessed were those believed to be associated with pathogenic bacterial contamination of broiler chickens from transport to market, to sale to consumer. The questionnaire contained 42 questions grouped under the headings; Sociodemographic data (seven questions), Questions on hygiene practices during transport, slaughter and display (20 questions), Knowledge about food-borne illness and *Campylobacter* spp. in meat (seven questions) and Questions on attitude (eight questions). The questionnaire was written in English and pre-tested before being used. All data was collected by the same person (the main author) in the local language. The interviews were conducted immediately after the purchase of the chicken carcasses, and responses were recorded on paper. The questionnaire is included as supplementary material.

The vendors were informed about the study, including that participation was voluntary and anonymous, and that they could withdraw at any time. They were recruited on the same day data collection was conducted, which took place from February 1^st^ to August 8^th^, 2022. Written consent was obtained from each vendor.

The study protocol was approved by the School of Biosecurity, Biotechnical and Laboratory Sciences, the College of Veterinary Medicine, Animal Resources and Bio-security (COVAB), Makerere University (reference: SBLS/HDRC/21/09) and by the Uganda National Council of Science and Technology (approval number: A156ES), before the study was conducted.

### Bacterial isolation and identification

The analyses were initiated within one hour after arrival at the laboratory and performed according to ISO 10272–1:2017 [32], with some modifications. Briefly, each carcass was rinsed with 200 ml of Buffered Peptone Water by shaking for one minute in a new, clean polythene bag. The bottom corner of the plastic bag was sanitised with 70% ethanol and aseptically cut. Ten ml of carcass rinse was added to 90 ml of Preston enrichment broth in a new, clean stomacher bag and incubated at 41.5°C for 24 hours under microaerobic conditions using Campygen^®^ (Oxoid, Basingstoke, UK). After enrichment in Preston broth, two loopfuls (approximately 40 μl) of the sample were spread on modified Charcoal Cefoperozone Deoxycholate agar (mCCDA) (Oxoid, Basingstoke, UK), and the plates were incubated at 41.5°C for 48 hours under microaerobic conditions. After 48 hours of incubation, the mCCDA plates were examined for typical *Campylobacter* spp. colonies (greyish, metallic sheen, and flat, moist with a tendency to spread). Suspected *Campylobacter* spp. colonies were re-cultured on blood agar (National Veterinary Institute, Uppsala, Sweden and COVAB, Kampala, Uganda) and incubated at 41.5°C for 48 hours under microaerobic conditions to get pure cultures. To verify the reliability of the microaerobic environment and the overall experimental setup *Campylobacter jejuni* CCUG 43594 was included as a positive control in all anaerobic jars. This ensured that the growth conditions provided were optimal for *Campylobacter* spp. The colonies were confirmed based on morphology and motility examined under a Phase Contrast microscope (Zeiss, Oberkochen, Germany) and oxidase test (Nutriselect^®^, Lyon, France). One pure colony from each sample was streaked on blood agar and incubated in aerobic conditions at 37°C for 48 hours, and no growth confirmed that the suspected colony was *Campylobacter* spp. All confirmed *Campylobacter* spp. isolates were stored at -20°C in Brain Heart Infusion (BHI) broth (CM1135; Oxoid) with 15% glycerol.

### PCR confirmation of isolates

DNA extraction was performed using a DNeasy^®^ Blood & Tissue kit (QIAGEN, Sollentuna, Sweden) according to the manufacturer’s instructions. Extracted DNA was stored in Eppendorf tubes and kept at -20°C before transport to the microbiology laboratory at the Department of Animal Biosciences, Swedish University of Agricultural Science, Uppsala, Sweden, where the tubes were kept at -20°C until analysis.

Multiplex PCR was performed to confirm that isolates belonged to the genus *Campylobacter* spp. and for species identification (*C*. *jejuni*, *C*. *coli*, *C*. *lari* or *C*. *upsaliensis*) using specific primers for 23S rRNA, gene *hip*O, *gly*A, *cpn*60 and g*ly*A, respectively [33, 34]. A known strain of *C*. *jejuni* (ATCC 3350) was used as positive control and pure distilled water as negative control. PCR amplification was performed using One Taq DNA polymerase (QIAGEN, Sollentuna, Sweden) in a reaction volume of 15 μl, comprising of 0.25 μl forward and reverse primers, 7.6 μl of One Taq 2X Master Mix, 1.25 μl of nuclease-free water, two μl of the DNA template. PCR amplification conditions comprised of an initial denaturation at 95°C for 15 minutes, followed by 30 cycles of denaturation at 94°C for 30 seconds, annealing at 57°C to 62°C for 90 seconds, and extension at 72°C for 90 seconds and one cycle of final extension at 72°C for 10 minutes. The PCR products (5 μl) and DNA ladder were loaded and electrophoresed in a 1.5% agar rose gel in 1x TAE buffer containing Gel Red at 110 V for 60 minutes. Electrophoresed amplicons were visualised by ultraviolet transillumination and documented using the Chemidoc XRS+ Gel imaging system.

### Antimicrobial susceptibility testing

Antimicrobial susceptibility tests (AST) were performed on pure isolates from all isolated *Campylobacter* spp. strains, including the antimicrobials tetracycline (Tet) class tetracycline, ciprofloxacin (Cip) class fluoroquinolones, streptomycin (Strep) class aminoglycoside, erythromycin (Ery) class macrolid and nalidixic acid (Nal) class quinolone using the disc diffusion test method according to European Committee of Antimicrobial sensitivity testing (EUCAST). The included antimicrobials were selected as they were commonly used in the livestock sector in Uganda. Briefly, a suspension of *Campylobacter* spp. in sterile saline to the density of 0.5 McFarland turbidity was evenly spread on Muller Hinton agar supplemented with 5% (v/v) defibrinated horse blood and 20 mg/L b-NAD (Muller Hinton Fastidious (MHF) agar) (SVA, Uppsala Sweden). The antimicrobial discs (Oxoid, Basingstoke, UK) of the antimicrobials mentioned above were placed on MHF agar using a disc dispenser and incubated at 41.5°C under microaerobic conditions. The growth inhibition zones were measured after 24 and 48 hours and interpreted according to the standards of EUCAST (www.eucst.org/clinical_breakpoints). Since no validated cut-off values by disc diffusion test for *Campylobacter* spp. regarding resistance to streptomycin and nalidixic acid existed when this study was performed, only no growth inhibition (0 mm) was determined as resistant. Multidrug resistance was defined as resistance to three or more antimicrobial classes [35]. This means that resistance to the quinolones ciprofloxacin and nalidixic acid was considered resistance to one antimicrobial class.

### Statistical analyses

All questionnaires were checked for consistency on the same day as data collection, coded and entered in Microsoft Excel. Comparisons of categorical variables, with the dependent variable being the occurrence of AMR *Campylobacter* spp., were performed using Chi-square and Fischers test for all variables included in Table 4 with the following modifications: for the variable ‘Source of birds’ comparison was made between farms within and outside the district; for the variable ‘What is used for cleaning slaughter surface’ comparison was made between ‘Clean water’ and ‘Clean water with detergent’; for the variable ‘Display method’ comparison was made for ‘Hang in open’ and ‘Others’. A p-value <0.05 was considered significant. Analysis was performed using the Social Science Statistics (www.socscistatistics.com/tests/).

## Results

### Prevalence of *Campylobacter* spp.

One broiler chicken carcass was purchased from each of four vendors at each market. There were, thus, 40 samples per district, making a total of 120 broiler chicken carcass samples. *Campylobacter* spp. were isolated from 79 (66%; 95% Confidence Interval, 65.6–66.1%) of them. The prevalence of *Campylobacter* spp. in broiler chicken carcass rinse samples in Kampala, Mukono, and Wakiso districts were 80%, 53% and 65%, respectively. All 79 isolates were confirmed as *Campylobacter* spp. by PCR. Of those, 32% (n = 25) were *C*. *jejuni* and 14% (n = 11) *C*. *coli*. The remaining 54% (n = 43) comprised a mixture of *C*. *jejuni* and *C*. *coli* (Table 1).

**Table 1:** Distribution of *Campylobacter* spp. isolated from 120 broiler chicken carcass rinse samples collected in three urban districts in Uganda.

	Kampala (n = 40)	Mukono (n = 40)	Wakiso (n = 40)	Total (n = 120)
***C*. *jejuni***	4	14	7	25
***C*. *coli***	4	3	4	11
***C*. *jejuni* and *C*. *coli***	24	4	15	43
**Total**	**32 (80%)**	**21 (53%)**	**26 (65%)**	**79 (66%)**

### Distribution of antimicrobial resistance

AST results were performed on the pure cultures of *C*. *jejuni* (n = 25) and *C*. *coli* (n = 11). All *C*. *jejuni* isolates were resistant against at least two of the tested antimicrobials; all showed resistance to tetracycline, 22 (88%) to ciprofloxacin and seven (28%) to erythromycin. Among the eleven *C*. *coli* isolates, nine (82%) showed resistance to tetracycline, and eight (73%) were resistant to erythromycin, ciprofloxacin and nalidixic acid. Multi-drug resistance (i.e. resistant to at least three different antimicrobial classes) was observed in eight (32%) *C*. *jejuni* isolates and five (45%) *C*. *coli* isolates (Table 2).

**Table 2:** Distribution of antimicrobial resistance among 11 *C*. *coli* and 25 *C*. *jejunii* isolated from broiler chicken carcasses collected from three urban districts in Uganda. Dark grey rows indicate multidrug-resistant (MDR) isolates, i.e. resistant to at least three different antimicrobial classes (ciprofloxacin and nalidixic acid belong to quinolones). Lighter grey rows indicate MDR *Campylobacter* spp. isolates that showed intermediate resistance (I) against ciprofloxacin.

***C*. *jejuni resistance***	**Kampala**	**Mukono**	**Wakiso**	**Total**
Cip+Nal*+Tet+Strep*		2		2
Cip+Nal*+Ery+Tet		1	2	3
Cip(I)+Nal*+Ery+Tet	1			1
Cip+Ery+Tet		1		1
Cip+Tet+Strep*			1	1
Cip(I)+Ery+Tet	1		1	2
Cip+Nal*+Tet	2	9	1	12
Cip+Tet		1	2	3
***C*. *coli resistance***	**Kampala**	**Mukono**	**Wakiso**	**Total**
Cip+Nal*+Ery+Tet+Strep*	1	1		2
Cip+Nal*+Ery+Tet	2		1	3
Cip(I)+Ery+Tet			1	1
Cip+Nal*+Tet		2		2
Cip+Tet	1			1
Cip(I)+ Nal*+Ery			1	1
Cip(I)+Ery			1	1
**Total**	**8**	**17**	**11**	**36**

*No validated cut-off value exists; therefore, no growth inhibition (0 mm) was deemed resistant.

### Socio-demographic characteristics

All vendors from which the broiler chicken carcasses (n = 120) were obtained were interviewed. The majority of them (87%) were men, 31 to 40 years of age and had either secondary (46%) or primary level (36%) education (Table 3). Forty-eight per cent of the vendors had been in business for ≥ 5 years and about half of them ran their own business or were employed (each around 48%).

**Table 3:** Socio-demographic characteristics of 120 vendors included in a study investigating food safety practices and knowledge, and prevalence of *Campylobacter* spp. in broiler chicken carcasses sold at urban informal markets in Uganda.

Variable	Category	No. of vendor
**Sex**	Female	16 (13%)
	Male	104 (87%)
**Age**	21–30	39 (32%)
	31–40	54 (45%)
	41–50	21 (18%)
	>51	6 (5%)
**Level of education**	No formal education	17 (14%)
	Primary	43 (36%)
	Secondary	55 (46%)
	Tertiary	5 (4%)
**Length of doing business**	6 months	11 (9%)
	1 year	14 (12%)
	2 years	29 (24%)
	3 years	18 (15%)
	5 years	48 (40%)
**Ownership of business**	Owner	57 (48%)
	Hired	57 (48%)
	Co-shared	6 (4%)
**Member of a poultry association**	Yes	10 (8%)
	No	110 (92%)

### Food safety knowledge and practices

The majority (80%) of vendors obtained the broiler chickens from farms within their district, and live chickens were mainly transported using metallic cages; chickens from different farms were usually (62%) not mixed and most (96%) vendors did not withdraw feed nor water before slaughter (Table 4). Most of the broiler chickens (87%) were slaughtered manually at the markets, and 68% of the vendors cleaned the slaughter surfaces once a day, primarily using only clean water (63%). The utensils and chopping boards were most often cleaned once a day (68%), and 88% used water and detergent. Over half the vendors displayed the slaughtered broiler chicken carcasses in the open. Eighty-eight per cent of the vendors washed their hands before slaughtering the chickens and none had access to any cooling facility.

**Table 4:** Variables related to food safety practices included in a study investigating the prevalence of *Campylobacter* spp. among broiler chicken carcasses sold at urban informal markets in Uganda.

Variable	No. of vendors	Occurrence of *Campylobacter* spp.	
	(%)	Yes	No
**District**			
Mukono	40 (33%)	21	19
Wakiso	40 (33%)	26	14
Kampala	40 (33%)	32	8
**Source of birds**			
Farms within the district	95 (80%)	63	32
Farms outside district	23 (19%)	14	9
Other, e.g., other retailer^1^	2 (1%)	2	0
**Means of transport**			
Metallic cages	100 (83%)	70	30
Plastic crates	7 (6%)	4	3
Sacs	6 (5%)	3	3
Other (wooden cages, pick carrier)	7 (6%)	2	5
**Mixing birds from different farm**			
Yes	45 (38%)	28	17
No	75 (62%)	51	24
**Withdraw feed before slaughter**			
Yes, within 2–8 hours	5 (4%)	2	3
No	115 (96%)	77	38
**Withdraw water before slaughter**			
Yes, within 2–8 hours	4 (3%)	2	2
No	116 (97%)	77	39
**Place of slaughter**			
Outside market	16 (13%)	8	8
At the market	104 (87%)	71	33
**Frequency of cleaning slaughter surface**			
Once a week	11 (9%)	10	1
Twice a day	27 (23%)	17	10
Once a day	82 (68%)	52	30
**What is used for cleaning slaughter surface**			
Clean water	76 (63%)	51	25
Clean water with detergent	19 (16%)	15	4
Wastewater	5 (4%)	4	1
Dry clean with towel, brush, steel wire	20 (17%)	9	11
**Frequency of cleaning utensils and chopping board**			
After each usage	23 (19%)	15	8
At the end of the day	97 (81%)	64	33
**What is used for cleaning utensils and chopping board**			
Clean water and detergent	106 (88%)	71	35
Wastewater	5 (4%)	3	2
Dry clean with towel, brush, sponge	7 (6%)	4	3
Clean hot water	2 (2%)	1	1
**Display method**			
Hang in open	78 (65%)	56	22
Other:	42 (35%)	23	19
Hang inside glass	9 (21%)	4	5
Basins, buckets and saucepans	27 (64%)	14	13
Slaughter on order	6 (15%)	5	1
**Refrigerate excess meat**			
Yes	23 (19%)	14	9
No	97 (81%)	65	32
**Wash hands before slaughter** ^1^			
Yes	105 (88%)	72	33
No	15 (12%)	7	8
**Wash hands with soap** ^1^			
Yes	98 (82%)	66	32
No	22 (18%)	13	9

^1^ Self-reporting

Analyses showed there was a difference (p = 0.03) in the prevalence of *Campylobacter* spp. between the three districts and a tendency that broiler chicken carcasses hung in the open were associated with findings of *Campylobacter* spp. from the carcass rinse (p = 0.06).

Slightly more than half (n = 67) of the vendors had heard about food-borne illnesses, and the most common symptoms mentioned were headache and vomiting. None of the vendors had heard of *Campylobacter* spp. or knew its significance. Most vendors knew about hygienic practices to prevent foodborne illness and were willing to change practices if they were wrong (97%) (Table 5).

**Table 5:** Food safety knowledge among 120 vendors selling broiler chicken at informal urban markets in Uganda.

Correct statements	No. of vendors that agreed with the correct answer
Meat from hygienic sources is safe to eat	64 (53%)
Fresh and old meat should not be kept together	81 (68%)
Soap is necessary when washing hands	91 (76%)
Hand hygiene prevents food-borne illness	87 (73%)

## Discussion

This study showed a high prevalence (66%) of *Campylobacter* spp. in broiler chicken carcasses sold at urban informal markets in a low-income setting, highlighting the importance of this bacteria from a public health perspective. There are relatively few published studies on *Campylobacter* spp. at the retail level, including informal markets and supermarkets, in LMICs. However, our findings are consistent with results from other African studies investigating the occurrence of *Campylobacter* spp. among chicken and chicken products at the retail level in Kenya and Egypt [16, 36]. Other studies from South Africa and Ghana reported *Campylobacter* spp. findings of 23% and 38% in raw chicken at the retail level [24, 37]. In the current study, slightly more than half of the samples contained a mixed culture of *C*. *jejuni* and *C*. *coli*. This finding could be due to the broiler chickens being colonised with a mixed culture of *Campylobacter* spp.; it could also be caused by cross-contamination of both species during processing by the vendors. The dominance of *C*. *jejuni* in the present study is in line with what has been reported in Europe and globally [1, 3].

Antimicrobial resistance is a serious One Health threat [38], and global data shows that the proportion of antimicrobials with more than 50% resistance has increased from 0.15 to 0.41 in the chicken population in LMIC [39]. In the present study, all or most of the *Campylobacter* spp. isolates (100% in *C*. *jejuni* and 82% in *C*. *coli*) were resistant to tetracycline, a commonly used antimicrobial in animal production in many countries [28, 39]. The results from the present study are consistent with findings from other studies conducted in Africa [25, 40]. We also observed a high resistance against ciprofloxacin (88% for *C*. *jejuni* and 73% for *C*. *coli*), which also agrees with previous studies [26, 40]. Due to the lack of validated cut-off values for resistance to nalidixic acid by disc diffusion testing in *Campylobacter* spp., resistance was defined by the absence of growth inhibition. Despite this criterion, almost three-quarters of the isolated *C*. *jejuni* and *C*. *coli* showed resistance to nalidixic acid. All isolates resistant to nalidixic acid were also classified as resistant or intermediate to ciprofloxacin. This result was expected, as both nalidixic acid and ciprofloxacin are quinolones, and mutation of T86I in the DNA gyrase (gyrA) gene of *Campylobacter* spp. is a common source of resistance to quinolones [41]. Interestingly, high resistance against erythromycin was detected, especially among *C*. *coli* (73%). However, this should be interpreted carefully due to the small number (n = 11) of *C*. *coli* isolates. Ciprofloxacin and erythromycin are critical to human medicine as resistance to the antimicrobial classes fluoroquinolones and macrolides have been increasingly observed. These antimicrobials are also commonly used to treat campylobacteriosis when clinical therapy is warranted [39]. Even if the number of isolates subjected to AMR testing in this study was relatively small, it is evident that there are very high levels of AMR, including MDR, among the *Campylobacter* spp. isolated from the broiler chicken carcasses. The resistance against nalidixic acid and streptomycin is probably higher than shown in our results since only no growth inhibition was deemed resistant in this study.

The high levels of AMR can most likely be explained by the widespread use of antimicrobials in the study area to prevent and treat diseases in poultry and as growth promoters [28]. The sale of antimicrobials in Uganda requires a prescription; however, in practice, most antimicrobials can be obtained over the counter from local drug outlets [29] and studies from Uganda and Kenya show that farmers often self-medicate chickens [28–30].

The high prevalence of *Campylobacter* spp. in broiler chicken carcass rinse samples in this study was most likely linked to slaughter contamination and poor food hygiene practices enabling transmission of pathogenic microbes. Poor sanitation and food hygiene practices when slaughtering and selling broiler chicken and products thereof are well-known risk factors for *Campylobacter* spp. transmission, especially in low-income settings [12, 37, 42–44]. Broiler chickens of slaughter age are known to carry *Campylobacter* spp. in their gastrointestinal tracts, and faecal matter can contaminate feathers and carcasses during catching, transportation and the entire slaughter process. Our study documented manual slaughtering and poor hygienic practices in the chicken handling facilities at the included informal markets, which is corresponding with results from a previous study investigating food safety practices at fast-food restaurants in Kampala, Uganda [45]. Furthermore, the non-withdrawal of feed and water prior to slaughter increases the risk of rupture of the intestines during processing and, hence, carcass contamination. It was also reported that only one-fifth of the included vendors cleaned utensils and surfaces after each usage, which is an important measure to prevent cross-contamination of pathogenic bacteria. The current study detected a variation of *Campylobacter* spp. prevalence between the included districts. This could be due to factors related to the management of the broiler chickens, differences in transport means to the market and variations in hygienic practices by the market vendors. No relevant risk factor for contamination was identified in the present study, possibly due to a relatively small sample size and poor hygiene practices throughout the entire production chain.

In the current study, only informal markets were included as these are where most people in the capital, Kampala, buy food. Food safety may vary between unregulated informal markets and regulated supermarkets; for example, it has been shown that food samples from informal markets yielded more *Campylobacter* spp. than those from supermarkets [24]. In the present study, a complete lack of knowledge about *Campylobacter* spp. among the included vendors was recorded and only slightly more than half of the respondents had heard about foodborne illnesses. Unsafe food handling by market vendors is associated with poor knowledge regarding hygiene practices and constitutes a risk of spreading pathogenic microorganisms. The obtention that most of the vendors were aware of the importance of using soap after visiting the toilet and that most of them washed their hands before slaughter showed a general knowledge about personal hygiene. There were, however, generally poor practices related to cleaning slaughter surfaces, utensils and chopping boards. The inadequate hygienic practices depict unsafe food handling, enabling the spread and cross-contamination of food-borne pathogens.

A relatively wide confidence interval for the sample size calculation was used to adapt the study to available resources. However, the results correspond with those reported in other regional studies [14, 16]. The vendors included in the study were not selected randomly, as obtaining an accurate list of vendors selling chicken at the included markets was impossible. Instead, we purposely selected vendors from different areas of the included markets. To improve food safety practices and prevent the spread and cross-contamination of pathogenic microbes, cost-effective and simple food safety interventions are needed, but these have largely been ignored in LMICs partly due to a lack of evidence on the burden of food-borne diseases [46]. However, it is promising that a large majority of the respondents in the current study showed willingness to change practices if needed. This finding is encouraging and calls for sensitisation and training of vendors on safe food handling practices to inhibit contamination by *Campylobacter* spp. and other foodborne pathogens. Short-term improvements in food hygiene practise as a result of training and capacity sharing have been documented, but creating an enabling environment that supports long-term outcomes is more challenging [46, 47]. Future research could focus on, for example, responsible antimicrobial usage and interventions to improve food safety at informal markets.

## Conclusion

This study highlights the widespread presence of Campylobacter spp. in broiler chickens sold at informal markets in an urban low-income setting, underscoring a public health concern. The high levels of antimicrobial resistance (AMR) detected in both C. jejuni and C. coli highlight the pervasive exposure of these bacteria to commonly used antimicrobials, likely driven by their un-restricted use in poultry production. Poor hygiene practices in slaughtering and handling, combined with limited vendor knowledge of foodborne illnesses, contribute to cross-contamination and the spread of Campylobacter spp. The lack of cooling facilities and inadequate cleaning routines exacerbate the risk of contamination. Despite these challenges, most vendors expressed willingness to improve food safety practices. This presents an opportunity for targeted interventions, including education and training, to mitigate contamination risks. Implementing simple, cost-effective strategies could play a crucial role in improving food safety and controlling the spread of AMR in Uganda. The results obtained from the present study can constitute useful information, especially for veterinarians, public health authorities and researchers operating in similar context.
